# Mindfulness and technostress in the workplace: a qualitative approach

**DOI:** 10.3389/fpsyg.2023.1252187

**Published:** 2023-11-13

**Authors:** Athina Ioannou

**Affiliations:** Surrey Business School, University of Surrey, Guildford, United Kingdom

**Keywords:** mindfulness, technostress, workplace, organisation, information technology

## Abstract

Information Technology (IT) has been vastly characterized as a double-edged sword, offering significant benefits to individuals but at the same time bringing certain negative consequences, such as technostress. Technostress can severely affect individuals in the workplace, causing fatigue, loss of motivation, inability to concentrate, dissatisfaction at work and reduced productivity among others; thus significantly affecting individual well-being work as well as increasing costs for organisations. Recently, studies have shown the beneficial role of mindfulness in reducing technostress experiences of individuals; however, the evidence that exists until today is very limited, and mostly focused on evaluating the impact of mindfulness on technostress and its negative consequences. As the current research stands, at the moment it is relatively unknown how mindfulness affects the underlying mechanisms of technostress experiences of individuals. Through semi-structured interviews with 10 knowledge workers, the current study explores how mindfulness alleviates technostress within the workplace, by investigating the experiences of more mindful employees and learning from their practices. Findings offer a deeper insight into the relationship of mindfulness and technostress, revealing a toolkit of the underlying strategies that more mindful and IT mindful individuals deploy as well as their perceptions during technostress experiences at work thus shedding light on the path between mindfulness and technostress. The study contributes both to academia and practice, offering important implications to managers and practitioners that strive to improve employee well-being within organisations.

## 1. Introduction

Information Technology (IT) has been vastly characterized as a double-edged sword (Maier, 2014). The power and advances of Information Communication Technologies (ICTs) have provided significant benefits to individuals and organizations; enabling them to analyse vast amounts of data while also facilitating flexibility to employees by creating mobile working environments diminishing geographic, time, location barriers (Yuan et al., 2013). While the proliferation of ICTs within the organizations has led to tremendous improvements in their performance and efficiency, those advances have come with certain costs. There has been increased interest in the negative aspects of ICT usage and especially on the stress caused by ICTs, called technostress. Technostress refers to the stress experienced by individuals in organizations due to the extended use of ICTs (Tarafdar et al., 2017). Symptoms of technostress include fatigue, loss of motivation, inability to concentrate, dissatisfaction at work and reduced productivity (Nastjuk et al., 2023) all of which are translated into huge monetary costs for organizations. It is estimated that workplace stress costs more than 1 trillion dollars every year to US businesses due to decreased employee productivity, absenteeism and turnover (Russo et al., 2021). As a result, it becomes apparent that technostress has a profound impact on business performance and the overall success of organizations, thus measures need to be taken to mitigate this phenomenon.

A wealth of studies has been published on the phenomenon of technostress. Most studies have focused on the factors that contribute to technostress, called technostress stressors, as well as investigating the impact of technostress on numerous organizational outcomes such as productivity, and job satisfaction, among others (Tarafdar et al., 2017). Recently, studies have indicated the important role of mindfulness in mitigating the negative consequences of technostress in organizations (Ioannou et al., 2022). Mindfulness is described as the idea of a dynamic, rich state of awareness, involvement and alertness, “a state of conscious awareness in which the individual is implicitly aware of the context and content of information” (Langer, 1992, p. 289). Evidence suggests that mindfulness practices can offer a wealth of benefits to individuals such as decreasing stress and anxiety, increasing mental clarity, improved memory and higher job satisfaction. Most of existing literature on technostress and mindfulness have focused on deploying quantitative studies, measuring the concepts through self-reported measures and surveys (Fischer and Riedl, 2019). Results of such studies have significantly enhanced our current understanding of the effects of mindfulness on technostress within the workplace and its negative consequences, as well as potential methods that can be used to alleviate technostress conditions. However, further research is deemed as essential, focusing on exploring the underlying conditions of technostress and how mindfulness counteracts such phenomenon through qualitative investigations, that have been in scarcity until today (Ioannou et al., 2022). Previous studies have attempted to examine factors that can alleviate the consequences of technostress, recommending organizational mechanisms such as literacy facilitation, technical support and involvement facilitation as means that can reduce the adverse impact of technostress conditions on individuals (Nastjuk et al., 2023). However, such investigations have merely tested the impact of such methods through quantitative surveys, while it still relatively unknown how such methods would work in real-life conditions. Since the problem continues to exist in today’s organizations, further research is deemed crucial in this matter. Addressing these calls for further research, the current study aims explore the role of mindfulness in alleviating the negative consequences arising from technostress within the workplace, by investigating the experiences of more mindful employees and learning from their practices. The present study contributes both to academia and practice, by evaluating the role of mindfulness in reducing technostress conditions that arise within the workplace offering important implications to managers and practitioners that strive to improve employee well-being within organisations.

## 2. Literature review

### 2.1. Technostress

During the last decade, there is a significant volume of published studies focusing on stress caused by ICTs in the work environment, a phenomenon also called technostress. Stress can disrupt the working environment and cause negative consequences in individuals such as poor individual performance, health problems and high absenteeism as well as poor decision making and communication problems (DeFrank, 2012).

The rapid advancement of technologies has created a significant difference between the knowledge that the employee currently possesses and the one needed by the ICT in use. Current ICTs create a sense of constant connectivity to individuals by extending the conventional workday through several ICT applications such as Internet, emails, mobile phones and instant messaging (Tarafdar et al., 2015). In addition, multitasking, IT interruptions and information overload caused by the constant usage of ICTs within the workplace, introduce a new way of working demanding a higher load of information to be dealt within a shorter amount of time. It becomes apparent that all previously mentioned situations create feelings to individuals of being unable to cope with technology thus leading to stress or else called technostress (Tarafdar et al., 2017). Occurrences of technostress happen due to the rapid changes in ICTs workplace, forcing individuals to continuously adapt to the changing physical, social, cognitive requirements impeded by ICTs use (Tarafdar et al., 2007). Technostress affects individuals on psychological, physical, behavioural and even biological level (Agogo and Hess, 2015). The symptoms that an individual may exhibit range from fatigue, inability to concentrate and frustration to loss of motivation, dissatisfaction at work and burnout (Ioannou et al., 2022). Click or tap here to enter text. According to World Health Organization, the costs of stress related outcomes within the workplace such as high absenteeism, productivity losses and increased employee turnover intention are estimated at 1 trillion dollars in the US industry every year, while in Europe respective costs come to around 240 billion euros (Russo et al., 2021). Therefore, it becomes apparent that technostress is a crucial issue for organizations that needs to be effectively addressed as it creates huge monetary and psychological costs both to businesses and individuals. These costs will continue to rise unless actions are undertaken that will moderate the consequences of this phenomenon (Russo et al., 2021).

The phenomenon of technostress has received significant attention during the last decade. Several studies have investigated the concept of technostress within various contexts and its impact on numerous organizational outcomes, such as productivity, job satisfaction (Nastjuk et al., 2023), organizational commitment (Maier et al., 2015) and workplace flourishing (Harunavamwe and Ward, 2022). Also, a number of studies have investigated the impact of moderating variables such as literacy facilitation, technical support and involvement facilitation (Ragu-Nathan et al., 2008; Tarafdar et al., 2010, 2015; Berger et al., 2023) as organizational mechanisms that can reduce the impact of technostress on individuals. Previous studies have examined the influence of personality characteristics on technostress (Srivastava et al., 2015) as well as the impact of information overload on technostress (Ayyagari, 2012). Furthermore, previous research has shown that high technology dependence increases the levels of perceived technostress on individuals (Shu et al., 2011). While most of the extant literature has examined the phenomenon of technostress in the context of general technology usage within the workplace (Ragu-Nathan et al., 2008; Tarafdar et al., 2010, 2015), more recent studies have focused on exploring the impact of technostress derived from emerging technologies such as artificial intelligence (Kumar et al., 2023), smartphones (Wang et al., 2023) and social networks (Maier et al., 2015). Moreover, in the growing body of technostress literature quantitative studies incorporating a survey-based approach with self-report measures are mostly dominant, while there is a surprising paucity of qualitative and multi-method research studies (Fischer and Riedl, 2017; Tarafdar et al., 2017).

The conditions that create technostress are called stressors. In their major study, Ragu-Nathan et al. (2008) identify and empirically validate the five conditions that create stress induced by the use of ICTs in the workplace and constitute in: techno overload, techno invasion, techno insecurity, techno uncertainty and techno complexity. ***Techno overload*** describes situations where ICTs force individuals to work faster and longer. Large amounts and high rates of information available through multiple ICTs create information overload, a situation where the individual cannot process efficiently the excessive loads of information within a short period of time, leading to feelings of stress and anxiety (Tarafdar et al., 2007). In addition, multitasking as well interruptions from multiple ICT applications pressure individuals to deal with several simultaneous tasks and incoming information thus creating tension and stress (Tarafdar et al., 2011). ***Techno invasion*** refers to situations where the individual feels always connected, never being free of technology and can be reached anywhere and anytime due to the use ICTs such as mobile phones, emails and messages (Tarafdar et al., 2011). As a result, the workday is extended and the individual feels being intruded in his private life thus exhibiting feelings of stress (Tarafdar et al., 2007, 2011). ***Techno insecurity*** describes situations where individuals feel threatened that they will lose their job either by other people who are more capable with new ICTs and possess better technological skills or by being replaced by new information systems (Tarafdar et al., 2010). ***Techno uncertainty*** indicates contexts where individuals feel unsettled due to the constant changes and upgrades of technologies inside the organizational workplace. Individuals need to continuously learn and educate themselves with new technology skills in order to use ICTs to complete their tasks. This constant re-learning and adaptation process creates stress to individuals as they continuously feel that their current skills are rapidly becoming obsolete (Tarafdar et al., 2007, 2010, 2011). ***Techno complexity*** refers to situations where individuals feel intimidated as well as inadequate in terms of technology skills due to the perceived complexity of newly introduced ICTs within the workplace. Individuals need to spend time and effort in order to learn how to use new complex systems and applications as well as deal with computer crashes and errors. As a result, feelings of stress and frustration arise (Tarafdar et al., 2010; Chandra et al., 2015). Most of the extant technostress literature has utilized the previously mentioned taxonomy of stressors in order to reveal their impact on numerous organizational outcomes such as productivity (Tarafdar et al., 2007), job satisfaction (Jena, 2015), organizational commitment (Kumar et al., 2013) and job burnout (Srivastava et al., 2015). In their interesting analysis Ayyagari et al. (2011) identify and analyse another set of similar stressors consisting of work overload, role ambiguity, job insecurity, work-home conflict and invasion of privacy. While a number of studies have utilized this latter classification of stressors (Maier et al., 2015), the majority of academic literature on technostress has investigated the phenomenon by employing the set of stressors initially proposed by Ragu-Nathan et al. (2008).

### 2.2. Mindfulness and technology

Mindfulness is described as a dynamic, rich state of awareness and observation of the present moment without reactivity or judgment (Glomb et al., 2011). It is described as the process of having attention to what is happening in the present moment, both internal and external stimuli, and observing them without judgement and assigning any meaning to them (Glomb et al., 2011). It incorporates the idea of being in the present moment (Langer, 1989). In contrast, mindlessness, the logical opposite of mindfulness, refers to a state of reduced attention accompanied by firm reliance and routine use of old categories, standard operation procedures, rigid decisions and inflexible thought processes (Braun and Martz, 2007).

A number of studies have explored the relationship of mindfulness with several IT related phenomena. Previous research has investigated the impact of IT mindfulness on mobile health technology continuance intention (Wu et al., 2022), the moderating effect of mindfulness on live streaming commerce (Gao et al., 2021), as well as its role in mobile payment adoption along with TAM (Technology adoption model; Flavian et al., 2020, Purbondaru et al., 2023). Other studies have examined the impact of mindfulness on Information Systems (IS) performance through top management support (Khan et al., 2013) as well as on ERP usage (Nwankpa and Roumani, 2014) and job performance in a mobile work environment (Dernbecher et al., 2014). Moreover, studies have combined mindfulness with dialectics in order to examine organizational reliability and IT capabilities (Carlo et al., 2012). Furthermore, studies have used mindfulness as a theoretical lens in order to investigate Agile Software Development (ASD; Mcavoy et al., 2013; Cram and Newell, 2016). Also, studies have argued that mindfulness influences IT dissatisfaction and re-invention (Nevo and Nevo, 2012) proposing the embedment of mindfulness in IS design in education (Wang, 2015). Previous studies have revealed that mindfulness can effectively mitigate the negative consequences arising from information overload (Wolf et al., 2011), increase individual performance in conceptual modelling (Bernárdez et al., 2018) and also alleviate post adoption regret arising from herd behaviour (Zou et al., 2015). Moreover, it has been shown that mindful adoption can increase perceived usefulness thus increasing task-technology fit at the post adoption stage leading to high satisfaction and continuance to use the technology (Sun et al., 2016). Also, in the context of IT security it has been empirically revealed that mindfulness, in the form of a training, can decrease individual ability to detect phishing attacks (Jensen et al., 2017).

In their seminal article (Thatcher et al., 2018), developed a domain specific individual-level measure of mindfulness and established the concept of IT mindfulness. They define IT mindfulness as a dynamic IT-specific trait, evident when working with IT, whereby the user focuses on the present, pays attention to detail, exhibits a willingness to consider other uses, and expresses genuine interest in investigating IT features and failures (Thatcher et al., 2018, p. 5). IT mindfulness, oriented in IT use and contexts, consists of four dimensions: alertness to distinction, awareness of multiple perspectives, openness to novelty and orientation in the present. Alertness to distinction refers to the extent that a mindful individual understands the capabilities of IT applications and the context that they will prove more useful. As a result, when the individual notices discrepancies between his use and the actual potential of the system or application, he is able to generate new ways of using the system (Thatcher et al., 2018). Awareness of multiple perspectives refers to the mindful individual who is able to identify and create multiple uses of a specific IT application as well as develop innovative solutions to problems that may arise in the working environment (Thatcher et al., 2018). Openness to novelty refers to the willingness of an individual to explore more potential and novel applications of the deployed system as he is always curious and flexible to experiment with the features of the system. At last, orientation in the present refers to the mindful individual who is involved as well as focused on the present moment and current context and able to adapt technologies at several different context (Roberts et al., 2007).

IT mindfulness constitutes a distinct concept than mindfulness; although the two concepts share the present moment orientation and awareness in the behaviour of an individual, they are different in their focus. Mindfulness refers to exhibiting mindfulness broadly, across various situations and times, during several contexts of everyday life whether at work or at home. On the other hand, IT mindfulness is an IT specific trait, describing the behaviour of an individual in specific situations and contexts. IT mindfulness is evident only when one is working with technology and oriented in the IT context. As a result, one person can be generally mindful but not necessarily demonstrate high levels of IT mindfulness. In their study (Thatcher et al., 2018), empirically revealed that IT mindfulness discriminates with mindfulness exhibiting more influence on IT related outcomes in post adoption system use. The concept of IT mindfulness has received research attention from very few theoretical research studies till today, aiming to investigate the impact of IT mindfulness on personality traits, including IT mindfulness, on technology induced stress (Maier et al., 2017). As a result, it becomes apparent that further research is deemed as crucial empirically investigating the concept of IT mindfulness, as existing academic research on the concept is still in its infancy.

### 2.3. Mindfulness and technostress

There is a considerable body of research investigating the effects of mindfulness in reducing workplace stress (Shonin and Van Gordon, 2015; Bonde et al., 2023; Michaelsen et al., 2023). While other studies have examined the beneficial effects of mindfulness on employee well-being, as increasing productivity, and improving job performance, reducing turnover intention and absenteeism (Good et al., 2016; Michaelsen et al., 2023). Till today, there are only a few studies focusing on the investigation of the relationship between mindfulness and technostress (Pflügner et al., 2021). Previous studies have highlighted that further research is essential in this area (Tarafdar et al., 2017). The limited evidence base that does exist, has shown that mindfulness can decrease job burnout through technostress in knowledge workers (Pflügner et al., 2021), while IT mindfulness, oriented toward IT use, can lessen perceptions of technostress (Ioannou and Papazafeiropoulou, 2017; Ioannou et al., 2022). While these studies have been mostly quantitative, survey-based examinations using self-reported measures for the measurement of mindfulness and technostress; qualitative investigations exploring the underlying mechanisms of mindfulness that affect technostress experiences are currently missing. Consequently, there is a need for additional studies exploring in more depth how mindfulness can act as a potential alleviator to stress induced by extended IT usage.

## 3. Methodology

In the current study, the data collection included semi-structured interviews with 10 knowledge workers. The overall aim of the study was to explore in depth the relationships of mindfulness and IT mindfulness with technostress stressors, and more specifically investigate how mindfulness affects each one of the stressors. The study aimed to explore the personal experiences of individuals during ICT induced stress conditions. Participants were recruited using convenience-based sampling as well as snowball sampling. An initial group of participants were sent the online survey at first, asking them to forward it to their colleagues as well. The current study was part of a larger project, thus participants were recruited in the first phase of the study either directly via email or through professional social media (e.g., LinkedIn). The targeted population of the study was set as working individuals that use technology at work daily, or as referred in the academic literature as “knowledge workers” in any industry. Knowledge workers are defined as employees involved with tasks that are more mental than physical; they perform complex tasks, including production and process of information. Knowledge workers are usually associated with high technology, business and information services sector (Brinkley et al., 2009). Since the current study aimed to investigate technostress within the workplace, knowledge workers were selected as the most relevant target population.

Interviews were deployed aiming to obtain more detailed information and detailed descriptions of employees’ personal experiences of technostress in the workplace. The study aimed to understand how these individuals react and cope with events that are triggered by technology usage within the workplace environment.

The interviews reached data saturation at the 10th interview so it was determined to stop the data collection at that point. Interviews constitute one of the various qualitative methods that one can use in order to reach data saturation. Data saturation is achieved when the researcher notices no new data, no new themes and no new coding from the undertaking of the interviews (Vasileiou et al., 2018) thus it is determined that the gathered sample is adequate enough to proceed to data analysis. In the current study, data saturation was achieved at the 10th interview thus the sample size being 10 participants.

An interview protocol was developed, based on extant academic literature on the concepts of technostress, mindfulness, and stress within the workplace as well as extant literature (Ioannou et al., 2022). The interview questions were focused on uncovering: (1) the position and job of the respondent as well as his daily work routine, (2) how comfortable, or else computer literate, the respondent is with technology in general while also understand his technology usage at work, (3) stressing situations that the respondent has experienced at work caused by technology. In addition to these questions, four scenarios were described to the respondents aiming to reveal and capture their coping strategies and reactions to stressors and specifically to the four technostress stressors, namely techno overload, techno invasion, techno complexity and techno insecurity (Tarafdar et al., 2007). For each one of the stressors, the respondent was presented with a scenario and was asked to describe a similar situation that they have encountered at work providing details about their feelings at the time as well as their reactions, and how they dealt with and resolved the ICT stressful situation. Through these scenarios, we aimed to uncover respondents’ experiences of ICT induced stress and more importantly their reactions and coping mechanisms with each one of the technostress stressors (see Appendix for the four scenarios). Before the beginning of each interview, each participant was asked to fill in a two-page questionnaire, aiming to assess their levels of mindfulness and IT mindfulness. The tests were used as a means of mindfulness assessment. In that way, by combining the level of mindfulness of each respondent with their responses to the stressors’ scenarios we can understand in more depth the relationship between mindfulness and technostress.

In this study, we adopted the Mindful Attention Awareness Scale (MAAS; Brown and Ryan, 2003) for the assessment of the mindfulness levels of the targeted individuals, as a relatively short scale was required for the purposes of the study as well as we were interested to capture a general mindfulness score. MAAS has been characterized as one the most widely accepted and used measurement scales in extant literature while also has been validated and received strong support by numerous studies and research contexts thus providing increased confidence to the research study. Consisting of 15 items, the MAAS scale measures mindfulness including a six-point scale rating the frequency of occurrence of every experience from Almost Always (1) to Almost Never (6). Example items include: “could be experiencing some emotion and not be conscious of it until sometime later” and “It seems I am running on an automatic without much awareness of what I’m doing.” Also, for the construct of IT mindfulness (ITM), we adopted the developed instrument from (Thatcher et al., 2018). As we aimed to assess IT mindfulness levels of individuals taking part in study, this study adopted the short version of the IT mindfulness scale that has already been validated by previous studies (Thatcher et al., 2018). The short scale consists of four (4) items each one measuring the four dimensions of IT mindfulness: Alertness to distinction, Awareness of multiple perspectives, Openness to novelty and Orientation in the present. Example items include: “I am very creative when using this technology” and “I like to figure out different ways of using this technology.” A five-point Likert scale was used ranging from Strongly Disagree to Strongly Agree.

As depicted in Table 1, the range of MAAS scores was 3–6 showing a moderate to high level of mindfulness, while the range for IT mindfulness (ITM) was 2.75–4 revealing a moderate to high level of IT mindfulness of the respondents. The range of values in both mindfulness scales show that all interviewees participating in the semi-structured interviews are characterized as moderately mindful and IT mindful individuals. The diversity of the sample was achieved by including a variety of occupations and job positions. All interviews were tape recorded and transcribed.

**Table 1:** MAAS and ITM scores of interviewees.

Id	Job description	Gender	MAAS	ITM
#1	Architect	F	3	3.75
#2	Marketing executive	F	4	3
#3	IT support	M	4.3	3.75
#4	Insurance executive	F	3.2	2.75
#5	Accountant	M	4.2	4
#6	Business analyst	F	6	4
#7	University lecturer	M	4.3	4.75
#8	IT advisor	F	3.3	3.25
#9	Management consultant	M	3.2	2.75
#10	Social media manager	F	4.8	3.5

Regarding the analysis, the current study deployed thematic analysis. Thematic analysis is a prestigious and widely used method deployed in academic research in a number of disciplines, such as psychology, aiming to encode qualitative information into explicit “codes” that describe the collected data as well as interpret the investigated phenomenon (Boyatzis, 1998). It is defined as a process “for identifying, analyzing and reporting patterns within data” (Braun and Clarke, 2006) that can be used by early researchers as it is rather accessible and relatively easy to understand, learn and use. According to Braun and Clarke (2006), there are six stages: familiarization with the data by transcribing and re- reading the data, and generation of initial codes, by identifying patterns, across the entire set of data takes place. Next, the searching for themes in the data takes place by grouping the previously generated codes in the interviewees’ responses. The review of the candidate themes follows accompanied also by their definition and naming. The sixth and last step of thematic analysis encompasses the final analysis, write up and presentation of the results. In the current study, the six phases (steps) of conducting thematic analysis were followed, as well as a theory-driven code development approach by firstly generating some overarching themes from existing literature and previous studies.

Moreover, in the current study, a deductive way of analysis of the qualitative data was followed by adopting a theory-driven code development based on mindfulness that has been adopted as the theoretical lens of the current study. As a result, the thematic analysis was guided by the theory of mindfulness for the development of codes and overarching themes. Moreover, in the current study, a latent interpretation of the gathered data, or else a latent level of analysis, was adopted going beyond the surface meaning of the data and seeking to understand the underlying ideas and assumptions that inform the content of the data (Braun and Clarke, 2006). The choice of the latent level of analysis was deemed as most suitable and appropriate for the current study; as we seek to gain insight into how mindfulness affects each one the stressors of technostress, inferences on mindfulness are needed to be made thus going behind the surface content of the collected data. The thematic analysis of the gathered data was conducted, by following the procedures recommended by Braun and Clarke (2006):

- **Step 1 (Familiarize with data)**: Transcription of the interviews from the audio recordings while at the same time ensuring to thoroughly read and re-read the data making notes for any potential interesting patterns that would be used to create initial codes.
- **Step 2 (Generate initial codes)**: The coding process on the collected data starts by identifying patterns and interesting pieces of information that would offer an interpretation of aspects of the investigated phenomenon (Boyatzis, 1998).The developed codes ranged from few words to maximum two lines. Furthermore, a manual procedure of coding was conducted, without using any particular software, since the amount of data allowed for a manual handling as well as offered the opportunity to immerse in more depth into the collected data.
- **Step 3 (Search for themes)**: During this stage, all the identified codes were organized into groups and tables in order to seek for potential overarching themes. Guided by the theoretical framework of the current study, the developed themes were then matched with the theoretical foundation of the study.
- **Step 4 (Review themes)**: The developed themes were reviewed and refined, ensuring that they are relevant both to the coded extracts and the whole data set.
- **Step 5 (Define and name themes)**: The themes were further refined by creating sub-themes; Furthermore, each theme was appointed with a title and a clear definition delineating the aspects of the data that it captures.
- **Step 6 (Produce analysis)**: By using the theory driven developed themes, the analysis was produced by choosing the most vivid examples and extracts representing the points that were deemed as essential to be demonstrated. The produced analysis was beyond a merely description of the data encompassing strong arguments towards the understanding of how mindfulness affects each one of the technostress stressors.

## 4. Findings

After careful analysis of the collected data, two overarching themes were identified in the data depicting: (1) the strategies that individuals are deploying during experiences of technostress in the workplace and (2) their perceptions during these experiences. Under these two overarching themes, several sub themes were identified that were categorized as more mindful and less mindful as depicted in Table 2. As a result, it becomes apparent that individuals deployed several mindful strategies as well as expressed mindful perceptions during their technostress experiences within the workplace. During the thematic analysis, strategies that more mindful and IT mindful individuals deploy during ICT stressed situations as well as their perceptions were revealed; some uncovered strategies are relevant to several stressors, such as prioritization deployed during overload and invasion situations while other revealed strategies, such as focus of attention, were relevant only in specific stressor situations. In Table 2, all revealed mindful strategies and perceptions and the respective stressors are depicted for summarization purposes. Strategies and perceptions are based on the definitions of mindfulness and IT mindfulness as have been discussed previously. Table 2 shows the themes that were identified during the thematic analysis of the interviews conducted with participants. The identified themes were then categorized in “strategies” and “perceptions” that more mindful and less mindful knowledge workers use during their daily encounters with technostress stressors in the workplace.

**Table 2:** Mindful strategies.

Themes			
Strategies		Perceptions	
Mindful	Less Mindful	Mindful	Less Mindful
Prioritization	Constant availability	Perceive as no threat	Stress induced perceptions
Focus of attention	Switching of attention		
Acceptance of situation as is			
Acting to resolve situation			
Update skills and knowledge			
Adaptation to different contexts			

**Figure 1:**
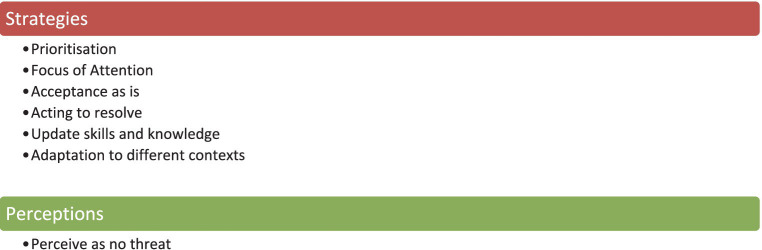
Strategies and Perceptions of more mindful and IT mindful knowledge workers.

In the sections that follow, the identified strategies and perceptions are described in detail one by one, accompanied by vivid quotes that were extracted from the interviews. However, it should be noted that all strategies are highly connected and interrelated with each other as they are considered as underlying mechanisms of the overall notion of mindfulness. The strategies and perceptions are discussed separately in each sub section for the sake of clarity and comprehension for the reader and we consider them not as separate entities but rather interdependent “forming” synergistically the construct of mindfulness (Figure 1).

### 4.1. Prioritization/constant availability

Prioritization refers to the evaluation of a group of items or tasks and the ranking of them in a particular order according to their importance and the priorities of the individual. The majority of the participants stated that during “techno – overload” situations, where incoming emails pop up, multiple interruptions and distractions occur, such as colleagues asking for help or clients are coming in the office while the individual is working on a task, prioritization of tasks was the primary deployed strategy in response to these situations. One of the participants clearly explained the notion of prioritization by mentioning that tasks considered as most important are ranked as first in order to be dealt with:

“*You need to prioritize and understand what is more urgent […] I prioritize the tasks and see what is more important*” (PC1, F, MAAS = 3, ITM = 3.75).

While another participant stated that the position of the person who is interrupting plays an important role:

“*Probably, it is a distraction in some respects, but it also depends on who it is and what position they are, where they are in the company … It is not so much a distraction as it is prioritization*” (PC5, M, MAAS = 4.2, ITM = 4).

Findings revealed that while individuals employ prioritization, they also take into account additional factors; depending on the urgency of the current task or matter, the importance of the current working task or as the position of the colleague as well as the elements of an incoming email, such as the subject, content and sender, the majority of the interviewees stated that interruptions will be ranked and dealt with accordingly based on defined priorities. One participant described that when several incoming emails pop up, he will first check the content of the email and determine the urgency of the matter, as well as the person who sent it and accordingly, he will apply prioritization of tasks. Especially when the current task is important, one participant described how he omits any incoming interruptions until he finishes the current task:

“*If I am in the middle of something that is quite important, then I will just ignore the incoming email until I finish. If it is just day to day work, …, and not actually in the middle of some complex operation, then as soon as a receive the email, I try to respond*” (PC3, M, MAAS = 4.3, ITM = 3.75).

As a result, findings revealed that by deploying prioritization of tasks individuals are able to effectively deal with “techno overload” situations thus remaining effective and productive at work. As all participants were assessed as moderately mindful, it can be inferred that prioritization constitutes an effective strategy of mindful individuals towards decreasing techno overload. By prioritizing competing tasks, more mindful individuals are able to adapt to the demands of each occurring situation, exhibiting resilience, focusing on the most important matters and feeling a sense of control over the ubiquity of ICTs in their work environment.

Furthermore, prioritization was reported from many participants as the deployed strategy when individuals experience “techno invasion” situations at the workplace. Technology can create blurring boundaries between work and personal life, with incoming emails, texts and other kinds of communication enabled by ICTs forcing individuals to be constantly available, outside the conventional work hours as well as during weekends and holidays. For some participants there are clear boundaries between personal and work life however a prioritization strategy outside of working hours is implemented depending on the urgency of the situation. One of the interviewees explains this notion by describing that during an emergency situation, such as the end of the month, the financial accountant might need help over the weekend if the system crashes, so the interviewee will check his email and respond only on this case:

“… *If something goes horribly wrong and the system crashes, I’ll get an email on Sunday. Now, I’ll check that, purely and simply because this is an emergency situation. So it’ll be that case where the Blackberry is ON, email from Mark? No, then, the Blackberry is off*” (PC5, M, MAAS = 4.2, ITM = 4).

While another participant described how he implements prioritization of communications during the weekend trying to put less than 100% of his efforts on the urgent situation:

“… *I try to put some efforts but not 100% over the weekend anyway*” (PC7, M, MAAS = 4.3, ITM = 4.75).

In contrast, less mindful individuals tend to be constantly available, mainly through their mobile phones, experiencing feelings of stress due to the constant connectivity enabled by ICTs rendering the boundaries between work and personal life blurry:

“*Stress, yes in terms of emails, you can always be reached by an email so even through your mobile or at your lunch break or everywhere that’s … stress*” (PC6, F, MAAS = 6, ITM = 4).

When participants were asked about their availability outside of work and whether they have time to unplug one participant mentioned feeling an inner obligation for availability and responding to emails:

“*I was trying to completely unplug, but if something is going on, it will still be in my mind…” “Sometimes, people do not really expect you to reply, but I feel that I need to, yes. I do not know why it is happening!*” (PC4, F, MAAS = 3.2, ITM = 2.75).

While another interviewee explained that he is always connected, with no boundaries existent, even during holidays, as he characterizes himself as a person that “likes to know what is going on”:

“*So, I’ve got my work email connected to my phone, so even when I am on holiday, I could turn the email on my phone off, but I do not*” (PC3, F, MAAS = 4.3, ITM = 3.75).

Overall, findings revealed that although most of the individuals receive emails outside of working hours, more mindful individuals deploy a prioritization strategy to deal with techno invasion. Varying their response depending on the urgency of the situation, mindful and IT mindful individuals effectively tackle feelings of techno invasion, as they define their own priorities and choose by themselves, instead of being forced by technology, when and under which circumstances they want to be available and contactable. On the other hand, less mindful individuals are more affected by techno invasion, feeling stressed from technology ubiquity as they are constantly available and contactable outside of working hours.

### 4.2. Focus of attention/attention switching

When individuals were asked how they respond to situations of multitasking with many incoming interruptions and occurring distractions (techno overload) while working on a task, many of them described that they focus their mental resources and attention on one task at a time, omitting any disturbing, unrelated information. One interviewee described that when a task is very important, any incoming email or task will be treated as an interruption:

“… *it depends on how much focus you need to put on what you are doing. If it is really something that you do not have to make mistakes and the task is very specific, then, you do not want to be disrupted. For example, in my previous job, if I was looking for a code bug (looking in the logs files), so I am looking for something so specific and I am doing comparison between files, then I do not want anyone to bother me*” (PC2, F, MAAS = 4, ITM = 3).

While another participant describes that he strives always to focus 100% of his resources on one task at a time by avoiding multitasking and instead prioritizing competing tasks by knowledgeably shifting his attention to the one that he considers as most important:

“*Usually, I am a person just focusing 100% on what I am doing, so when I am working on a paper, and somebody comes, then, I cannot do 50%*” (PC7, M, MAAS = 4.3, ITM = 4.75).

Despite working in a dynamic and constantly evolving environment, mindful individuals choose to focus their attention on one task at a time, able to not get distracted by unrelated tasks or interruptions. In contrast, less mindful individuals engage in multitasking, switching their attention from the main task at hand to other interruptions, thus causing vital information of the main task to be missed. For example, one participant mentioned that he uses a recording device during his client meetings, as he is performing multitasking almost every day thus, he gets distracted and crucial information can be missed:

“*So, like, I use this (recording machine) sometimes to record and when I am less busy with other clients, I listen to it if I need to prepare my report and I found it very useful, and if I do not have it, then, I will not be able to listen to them properly*” (PC8, M, MAAS = 3.3, ITM = 3.25).

As a result, it becomes apparent that more mindful and IT mindful individuals are able to focus their mental and physical resources on one task at time; preventing getting distracted from incoming interruptions occurring in the environment, as well as consciously shifting their attention to the matter that they value as most important at the present moment thus decreasing the impact of techno overload. However, less mindful individuals tend to lose their focus on the current working task by engaging in other tasks at the same time thus ending up missing vital information of the main task at hand.

### 4.3. Acceptance of situation as is

Acceptance of the situation as is refers to the strategy of an individual, when encountering a stressful situation caused by ICT usage, who acknowledges and perceives experiences with less negative emotions as well as accepts the idea that some things cannot change but instead we need to accept them as they are. When participants were asked about techno complexity experiences at work, referring to technology failures, errors and problems while working on a task, some participants stated that they are used to such situations and accept them as they are without experiencing any negative emotions:

“*So, (you) learn living with the technology*” (PC2, F, MAAS = 4, ITM = 3).

“*Things like that when you work in IT, they are everyday things*” (PC3, M, MAAS = 4.3, ITM = 3.75).

Another respondent explained that by accepting his own mistakes, in this case not saving his work on the computer thus having to repeat the task starting from scratch during software and hardware crashes, proved to be a beneficial experience to him, offering him space for personal growth, enhancing his self-competence and individual productivity:

“… *So, it does not bother me because I learnt to accept the fact that If I am the idiot and I do not save something, I know I can re-do it again quicker. If the system crashes and I lose a portion of my work, then I know that I can go back and get it quickly*” (PC5, M, MAAS = 4.2, ITM = 4).

By admitting his own mistakes, the respondent accepts the situation without feeling negative emotions or judgmental to himself thus being able to return back to re-do the task, evaluating this experience as having learnt something of value and accepting it as an opportunity for future growth.

Overall, the findings showed that mindful individuals respond objectively to technology failures and problems while working on a task (techno complexity) by accepting the occurring situation as is and feeling less negative emotions. As a result, it can be inferred that by accepting the situation as is, mindful individuals are left less depleted after an ICT stressful event thus are able to significantly decrease the impact of techno complexity.

Furthermore, acceptance emerged as a deployed strategy towards situations of techno insecurity. Individuals expressed positive perceptions and agreement with the notion that technology can replace their positions someday in the near future while at the same time they did not perceive it as an immediate threat:

“*Everything is possible with technology*” (PC7, M, MAAS = 4.3, ITM = 4.75).

Thus, it can be argued that mindful individuals feel less threatened by emerging technologies by accepting the possibility of getting replaced, not perceiving it as a negative event as well as being open to novel things and perspectives.

### 4.4. Acting to resolve situation

Findings revealed that in situations where technology failures occur during the workday such as computers crashing and applications running slow, several individuals responded by trying to find a solution and resolve the problematic situation either by asking help from IT support or by implementing workarounds or even both. One participant in particular explains that the delayed and ineffective service of IT desk led him into implementing workarounds:

“… *I went few times to the IT office downstairs. I went to the computer centre… when I know that somebody would contact me, then I ask them to send me emails to my personal email (Gmail account)*” (PC7, M, MAAS = 4.3, ITM = 4.75).

Another participant described that the first strategy when facing a technology crash is to attempt to fix it by own means and then resort to IT support:

“*We do have IT desk, so personally, I try to do it on my own first, because going to the IT desk might take some time… So, I try to fix it on my own, and if I cannot then, I have to ask for help from someone else*” (PC1, F, MAAS = 3, ITM = 3.75).

Findings showed that mindful and IT mindful individuals, feeling more confident as well as in control over problematic situations occurring due to ICTs, take the initiative acting towards resolving the occurring problems. Instead of being absorbed by habitual thoughts and feelings, such as frustration and stress, mindful and IT mindful individuals show eagerness to conclude their work tasks when problems occur, exhibiting innovativeness by seeking alternative and workaround solutions or resorting to the IT support department of the company, thus tackling the impact of techno complexity.

### 4.5. Update skills and knowledge

Most of the interviewed participants stated that they are not afraid of getting replaced either by emerging technologies or by other people as they strive to update their skills and knowledge by getting involved into new things and are eager to constantly evolve. One participant in particular clearly expresses this notion by highlighting also his feelings of certainty and control over technology:

“*My own aspect is that you need to be updated about what is happening. See what is happening around you… Technology cannot change me; I will change technology. So, you need to put yourself up to speed*” (PC8, M, MAAS = 3.3, ITM = 3.25).

Furthermore, many participants showed eagerness towards enhancing their skills and knowledge thus equipping themselves against the idea of becoming obsolete due to the constant updates and upgrades of organizational ICTs. Either through self-study or by attending organizational training programs, individuals seem to be very aware that they need to be up to date with technological advances in their domain. One participant explains the importance of attending training programs to stay ahead of colleagues, instead of falling behind and thus avoid risking her position in the company:

“*You cannot stay behind, otherwise you are out*” (PC2, F, MAAS = 4, ITM = 3).

While another participant delineates that his personal characteristics, such as innovativeness and curiosity, drive his eagerness towards self-studying in order to stay up to date in his domain:

“*I am very innovative. My course now (MSc), is really helping me in that. And, the PhD idea is part of this… Because, for you to be innovative, you need to rely on something, and make time for yourself to understand what is happening in the educational system, because nobody will just tell you this is what is going on*” (PC8, M, MAAS = 3.3, ITM = 3.25).

As a result, it becomes apparent that mindful as well as IT mindful individuals are willing as well curious to pursue learning activities towards updating their skills and knowledge thus “shielding” themselves from becoming obsolete in a dynamic and constantly evolving working environment filled with continuous upgrades in technologies and organisational ICTs. Thus, in this way IT mindful individuals combat the impact of techno complexity.

### 4.6. Adaptation to different contexts

A major theme that emerged in the collected data was the ability of participants to adapt to different contexts and more specifically to vary their response to the various occurring distressing situations, each time depending on the context and circumstances. Some of the respondents reported that in situations where technology and ICTs created feelings of stress they postponed their response and by taking a break. During an ICT stressful situation, a participant explains that a break from technology helps her towards tackling feelings of techno invasion:

“…*when sometimes I am so fed up, I will just go for a walk and leave it (phone) home. I need to leave it be away from me and then I am fine*” (PC2, F, MAAS = 4, ITM = 3).

While another participant described that by postponing his response, he can take a step back from the occurring situation and react more objectively. When incoming emails and information overload (techno overload) occur he acknowledges his current feelings and consciously takes a break before responding:

“… *so I am very careful how I respond to emails. Also, if it is something that has upset me or angered me, then I might give it a while and then respond…*” (PC3, F, MAAS = 4.3, ITM = 3.75).

The strategy of “taking a step back” is also deployed during technology failures at work (techno complexity) where an interviewee stated that he consciously steps away from a technology crash, while waiting for IT support to fix the problematic issue, accepting the occurring situation and without experiencing negative feelings:

“*I tend to go and make a coffee. If something is crashed, then, there is nothing I could do anything about it, so I sit back and fire up a ticket that say this needs to be fixed*” (PC5, M, MAAS = 4.2, ITM = 4).

By adapting to different contexts, and more specifically by varying their response depending on the context of the present moment, i.e., work or personal time, individuals decrease the impact of techno invasion. One participant vividly explains how he has created clear boundaries between work and personal life by limiting his availability outside of work settings, adapting to different contexts and unplugging from work when reaching home; thus decreasing the impact of techno invasion:

“*Yeah, generally it is a rule for me (to unplug) as soon as I step out the door… By the time it gets from work to home, I completely unwind. … My wife hates it. She hates the fact that I just unplug. Just switch off and go*” (PC5, M, MAAS = 4.2, ITM = 4).

Another participant describes that the severe effects of techno invasion can be mitigated by adapting her response to different contexts; for example, when on holidays she is considerably limiting her availability to work related interruptions:

“… *basically, you tend to work from the morning until the moment you go to bed. You do not have this 8 to 5 work time, so when I go to holiday, then that is off (the phone)…*” (PC2, F, MAAS = 4, ITM = 3).

As a result, it becomes apparent that mindful and IT mindful individuals are able to adapt to different contexts either by focusing on the present moment and varying their response, limiting their availability or taking a step back from the stressful situation and postponing their immediate response. Thus, they are able to react more objectively during the stressful occurring situation, experiencing less negative emotions and left less depleted. Thus, the impact of the stressor is decreased.

### 4.7. Perceive as no threat/habitual perceptions

Another major theme that emerged in the collected data was that interviewees did not perceive the stressful situation as a threat for any of the four techno stressors (overload, invasion, complexity, insecurity). More specifically, in situations of techno overload with multiple incoming interruptions while working on a task, a participant mentioned that she does not perceive the situation as threatening but rather as a challenge to work more and be efficient:

“*Sometimes when this happens I feel more happy because the situation triggers me*” (PC1, F, MAAS = 3, ITM = 3.75).

Likewise, during situations where ICTs allow constant connectivity out of working hours, participants described a similar perception; techno invasion was not perceived as a threat as individuals stated that they do not mind being contacted after working hours and they consider themselves flexible for client needs. Furthermore, during techno complexity situations findings also showed that participants accept the fact that technology failures happen sometimes, but these situations do not create unsettling feelings. At last, regarding techno insecurity, findings showed that most of the participants do not feel risk over getting replaced either by new technologies or by other people. Adding to that, most of the participants expressed an openness to new talents coming in the company, even if it involves individuals more enthusiastic with technology and equipped with more technological skills:

“*If you hire the right people, then they would affect things positively, and I think fresh people or fresh blood helps a lot…*” (PC7, M, MAAS = 4.3, ITM = 4.75).

Moreover, a very interesting notion was revealed from the analysis; The possibility that emerging technologies may replace people’s job positions is viewed rather as an opportunity for growth and move on to better, more interesting things than as a real threat:

*“… I look at that as being opportunity to move on to better things that are more interesting… So, it is not something that I consider to be a bad thing. I think there is something positive to come out of it*” (PC3, M, MAAS = 4.3, ITM = 3.75).

As a result, it can be inferred that mindful and IT mindful individuals perceive IT stressful events as less threatening without adding automatic and habitual negative appraisals. Being open to multiple perspectives and aware of the present moment situation, more mindful and IT mindful individuals can construct new categories, avoiding habitual thoughts and automatic reactions, thus not perceiving as a threat any of the stressors and decreasing the impact of technostress. In contrast, findings showed that perceptions of less mindful individuals differ significantly; Less mindful individuals appeared to experience more unsettling feelings during ICT stressful situations at work. Most of the participants mentioned that they have experienced feelings of stress, frustration, annoyance and anxiety during techno stress situations within the workplace. During techno invasion occurring events, individuals reported that they have experienced connectivity pressure, feeling being always on “standby” as well as great annoyance from the imbalance that technology invasion has created with their private life. Likewise, during techno complexity scenarios participants reported that technology errors and failures cause great amounts of stress and frustration as well as feelings of pressure to catch deadlines and finish their tasks on time and effectively. As a result, it becomes apparent that less mindful individuals tend to react more habitually, allowing the occurrence of automatic thoughts and reactions, being less able to combat the impact of the stressor thus experiencing distressing and overwhelming feelings from extended usage of ICTs.

## 5. Discussion and implications

Stress in organizations has been widely investigated in the academic literature in several disciplines such as Information Systems, Management and Organizational studies. Workplace stress has detrimental effects on employees’ health while at the same time causes severe negative socioeconomic consequences including reduced productivity, decreased job performance, higher rates of absenteeism and turnover intention, burnout and employee compensation claims (Shonin and Van Gordon, 2015) translating into huge monetary costs for organizations. A major source of stress within organizational settings is technology, as employees are obliged to utilize several different ICT applications in order to complete their work tasks. Technostress is described as the negative impact arising from ICT usage within the work environment and manifests in “emotional and physical stress associated with technology and the introduction of new technologies” (Meischke et al., 2015). New information and digital technologies have changed organizational settings as well as the workload of employees thus contributing to higher levels of stress. A considerable amount of literature has been published around the concept of technostress in the IS domain, however most of previous studies suggest the same three organizational mechanisms as means to reduce the negative consequences of technostress; literacy facilitation, technical support and involvement (Tarafdar et al., 2015). These mechanisms have become the main focus of extant studies in IS literature while there is a surprising paucity of research exploring further means that could alleviate the adverse aftereffects of technostress (D’Arcy et al., 2014). Moreover, the majority of extant technostress has been focused on quantitative investigations, while qualitative studies that explore in more depth the underlying dimensions of technostress and how mindfulness affects such phenomenon are surprisingly missing. As a result, the present research aimed to address these gaps by examining mindfulness as a technostress inhibitor or else a method to buffer the stressors that cause technostress, alleviate the adverse effects arising from extended ICT usage within organizational settings and ultimately contribute to employee well- being. Findings offer a deeper insight into the relationship of mindfulness and technostress, revealing the underlying strategies that more mindful and IT mindful individuals deploy as well as their perceptions during technostress experiences at work thus shedding light on the path between mindfulness and technostress.

Our findings reveal some of the strategies that mindful and IT mindful individuals deploy during ICT stressed situations; some uncovered strategies are relevant to several stressors, such as prioritization deployed during overload and invasion situations while other revealed strategies, such as focus of attention, were relevant only in specific stressor situations. Since that most of the identified strategies relate to more than one stressor, the following discussion will be structured per strategy, rather than per stressor, in order to illustrate the underlying mechanisms of mindfulness relative to each strategy and perception. There have been no similar studies in extant literature, investigating mindfulness and technostress stressors individually, that we can relate to and compare our findings. For this reason, we relate our results indirectly with existing mindfulness, stress and IS literature.

### 5.1. Mindfulness and technostress

The analysis of the semi-structured interviews revealed some of the strategies that more mindful individuals deploy during stressful situations at work as well as their perceptions during these experiences. In agreement with extant research, although the underlying mechanisms of mindfulness can be described separately, in reality they are working synergistically (Alberts and Hülsheger, 2015). Thus, for this reason, in this section we will discuss all identified mindful strategies and perceptions together.

Several previous studies have empirically shown that mindfulness can decrease the levels of stress that individuals experience at work (Michaelsen et al., 2023). Our findings support and extend previous research by revealing that mindfulness may decrease ICT induced stress that occurs within work settings. Our findings concur with previous literature arguing that mindfulness fosters more effective stress processing. More mindful individuals seem to cope with stress more effectively by using more adaptive strategies, such as direct dealing with the situation, acceptance and reinterpretation of the situation and less avoidant ways such as ignoring or escaping threatening stimuli (Ioannou et al., 2021) Direct dealing with the situation or else called active coping refers to direct actions of an individual to deal with the stressful situation. Evident in our findings, active coping, or else as we named the theme acting to resolve, was an emergent strategy in our findings deployed by individuals during technostress related situations; more mindful individuals put an effort and strived to resolve the distressing situation, when computers crashed or applications errors occurred, in order to be able to conclude their work tasks.

Prioritization of competing tasks and most important assignments is one of the primary strategies that mindful individuals deploy during situations where extended ICT usage creates stress at work (Shapiro et al., 2015). Fostering the ability to distance oneself from occurring stimuli, mindfulness allows room between impulse and reaction that an individual can utilize in order to notice distractions, prioritize and respond consciously and thoughtfully to demanding situations (Alberts and Hülsheger, 2015). Consistent with previous studies, our findings extend this notion by revealing that prioritization is a widely used strategy by mindful individuals during situations of techno overload and techno invasion at work.

Along with prioritization, focus of attention on one task at a time was found as a strategy when individuals were faced with information overload, situations demanding switching of attention and multitasking at work (techno overload). Our findings concur with previous studies demonstrating that mindfulness can decrease the negative effects of multitasking by increasing the average time that an individual spends on one task (Levy et al., 2012). Moreover, focusing on the IT context, Wolf et al. (2011) have empirically shown that mindful individuals can mitigate the negative consequences of information overload by focusing their attention on the relevant task at hand. The ability of mindful individuals to focus their attention intentionally on the current experience and omit any other incoming disturbing or unrelated information, but at the same time be aware of what is happening in the environment enables them to decrease the adverse aftereffects of techno overload situations. As a result, it becomes apparent that our findings, agreeing with extant literature, come as not surprising.

Moreover, taking a step back before reacting and responding to ICT stressful events was revealed as another strategy that mindful individuals deploy at work. Respondents in our study reported that during situations where technology and ICTs created distressing feelings, caused either by information overload (techno overload), technology invading personal life (techno invasion) or ICT applications crashing and producing errors (techno complexity), taking a break from the situation was the first resolution. Our findings are consistent with existing literature, explaining that the ability to take a step back from a situation is a major element of mindfulness lying upon the concept of “response flexibility.” Response flexibility occurs when an individual is able to take a step back and “slow down” before responding to any environmental stimulus (Glomb et al., 2011; Shapiro et al., 2015). Responding in a flexible manner gives the opportunity to the individual to carefully assess the situation before initiating any actions (Glomb et al., 2011). Previous studies have shown that mindfulness fosters reduced reactivity to occurring events as well as the ability to disengage and take a step back from distressing experiences by inhibiting automatic and habitual reactions; Thus, individuals are able to pause, reflect and consider thoughtfully how to react to workplace stressful events (Malinowski and Lim, 2015).

Our findings also showed that mindful individuals are more likely to accept a situation as it is, without experiencing overwhelming feelings or striving to change a stressful event occurring due to the usage of ICTs (techno complexity, techno insecurity). Existing mindfulness literature agrees with our results describing a mindful person as one who does not attempt to change any occurring experiences but rather observes what is happening at the present moment with openness, curiosity, acceptance and a non-judgmental attitude (Shapiro et al., 2015). As already mentioned before, mindfulness fosters more effective stress processing by using more adaptive strategies allowing acceptance and reinterpretation of the occurring situation (Ioannou et al., 2021).Our results showed that during situations where individuals are facing difficulties due to technology errors and crashing applications while working on a task, acceptance of mistakes was revealed as an underlying mechanism of their coping strategy. More mindful individuals accept their mistakes and are able to go back to their task without feeling negative emotions or being judgmental; thus acknowledging that they have gained something of value from this experience. According to existing literature, an individual who perceives mistakes from a mindfulness perspective is looking at the “silver lining” of the situation and is able to learn something of value, recognizing this experience as an opportunity for self enhancement and future growth (Carson and Langer, 2006).

A major theme that emerged from our findings was the fact that individuals did not perceive any of the technostress stressors as threats. Our findings were not surprising as they agree with previous mindfulness studies; More mindful individuals perceive stressful events as less threatening or demanding without adding automatic and habitual negative reactions (Ioannou et al., 2021).By creating a space between emotions and reactions, a mindful individual perceives stressful events as not threatening but rather as manageable (Schultz et al., 2015). Interestingly, the majority of the participants reported feeling fewer negative emotions during distressing experiences at work where technology failures occur (techno complexity), incoming emails pop up after office work hours (techno overload) or new technologies and talents are introduced in the organization (techno insecurity). Our findings support previous studies suggesting that mindfulness fosters the generation and prevalence of more positive and less negative emotions during difficult situations (Glomb et al., 2011; Good et al., 2016). At last, very interestingly our findings showed that some participants perceived ICT demanding situations as a positive challenge or else as an opportunity for personal growth. As mindfulness facilitates decoupling reactions from previous negative experiences, it allows room for pause and reflection so that the individual can re interpret the situation thus perceiving the stressor as a challenge that is beneficial rather than as a threat (Good et al., 2016).

Overall, our findings suggest a direct negative relationship between mindfulness; Able to cope with stress more effectively, mindful individuals have a wider range and more adaptive coping strategies during stressful situations thus being able to decrease the impact of technostress stressors within workplace settings.

### 5.2. It mindfulness and technostress

According to Thatcher et al. (2018) IT mindfulness is “a dynamic IT- specific trait, evident when working with IT” describing a user who is paying attention to the present moment and is willing as well as curious to experiment with the features of technology. IT mindfulness, oriented specifically in IT use and contexts, consists of four interrelated dimensions: Alertness to distinction, referring to the extent that a mindful individual understands the capabilities of IT applications and the context that they will prove more useful. Awareness of multiple perspectives referring to the mindful individual who is able to develop innovative solutions when problems arise in the working environment. Openness to novelty referring to the individual who is willing to explore more potential and novel applications of the deployed system as he is always curious and flexible to experiment with the features of the system. Orientation in the present referring to the mindful individual who is focused on the present moment and able to adapt his use of technologies at different contexts (Thatcher et al., 2018). IT mindfulness constitutes a rather under researched concept in IS literature; Firstly, introduced by Roberts et al. (2007), till today research focusing on IT mindfulness has been very limited (Maier et al., 2017, 2019; Thatcher et al., 2018; Ioannou et al., 2022).

Constant updates and upgrades of organizational ICTs as well as the emergence of new technologies create uncertainty and unsettling feelings to employees feeling they cannot keep up with the pace of new technologies, their skills are quickly becoming obsolete as well as fearing that they will lose their job. Our findings reveal that one of the major strategies individuals deploy towards combatting such distressing situations, techno complexity and techno insecurity instances, is the updating of their skills and knowledge. Extant research posits that IT mindful individuals are characterized as curious and open to new information and novel experiences while also open to intellectually challenging ideas. Also, IT mindful individuals are predisposed towards a novelty seeking behavior in use of IT as well as sensitive to their context; thus, strive to stay aware of new developments (Langer, 1989). A more IT mindful individual is continually searching for opportunities in the IT context that will help him to use technology more effectively in order to complete his work tasks. In agreement with extant literature, our findings revealed that IT mindful individuals are curious to learn new experiences and enhance their intellectual skills while also recognize the need to stay up to date with technological trends and advances in their respective domains; thus, they strive to enhance their skills and knowledge either on their own or by participating in organizational trainings.

Also, our findings showed that more IT mindful individuals act to resolve problematic situations that arise at work due to ICT failures or errors instead of staying inactive unable to continue their work tasks. As Carter et al. (2011) note, IT mindfulness is characterized by novelty seeking and novelty producing behavior in the use of IT, while “… a lack of [IT] mindfulness is consistent with a tendency to persist in using well-learned routines ….” As a result, IT mindful individuals are able to create innovative solutions (Thatcher et al., 2018) or even implement workarounds in order to achieve task technology fit. Moreover, more IT mindful individuals are not committed into certain ways of using technology, but instead are continually searching for opportunities that could improve their technology use when executing their work tasks; thus, they are flexible and able to adapt their technology use to dynamic and shifting environments and the context of each situation each time (Thatcher et al., 2018). In accordance with extant research, our results showed that during technology failures such as computers crashing or applications running slow that create obstacles and difficulties in the execution of work tasks (techno complexity), IT mindful individuals strived to seek solutions exhibiting novelty seeking and novelty producing behavior; either by resorting to IT support and in the meanwhile use alternative means to run their tasks or by applying alternative solutions, adapting their technology use to the current context and implementing “workarounds” in order to conclude their work tasks.

A major theme that emerged from our findings was that more IT mindful individuals were able to adapt to different contexts, sometimes also using prioritization at the same time. Our findings revealed that more IT mindful individuals can adapt to different contexts and vary their response and technology usage during techno invasion situations. During situations of incoming interruptions, such as emails occurring after office hours, during weekends or holidays, more IT mindful individuals were able to vary their technology use: By “unplugging,” limiting their availability and sometimes even defining priorities when being out of office, they were able to vary their technology use such as turning off the work mobile phone or using it only for personal circumstances thus creating clear boundaries between work and personal life. Our findings concur with existing research; According to Thatcher et al. (2018), IT mindfulness fosters sensitivity to different contexts and orientation in the present moment. IT mindful individuals are able to adapt their behavior to shifting environments, showing flexibility and resilience and becoming greatly involved in the current context. As they do not restrict themselves to pre committed ways of using technology they are able to adapt and thus vary their IT use depending every time on the current moment and their environment (Thatcher et al., 2018).

Moreover, our findings showed that more IT mindful individuals are able to focus their mental resources and attention on one task at a time, omitting any disturbing, unrelated information during situations of incoming interruptions, emails and occurring distractions (techno overload); However, they remain aware of their context and environment, with certain priorities defined. For example, participants mentioned that when a task is very important, they focus their resources on that but at the same time if something more important occurs they are ready to respond. Our findings come in accordance with extant research; IT mindful individuals are oriented in the present moment and their current IT context and technology use, focus on the immediate task at context and the specific situation but do not lose focus of stimuli outside the immediate task at hand (Thatcher et al., 2018).

Another major theme that emerged within our findings was that individuals may not perceive technostress stressors as significant threats. Especially during situations that induce techno insecurity, individuals responded with acceptance, expressing less negative feelings thus perceiving the stressor as no threat; acknowledging the dynamics of emerging technologies and accepting the possibility that technology may replace their job position in the future. Interestingly, for some participants, this possibility was not perceived as a negative event but rather as an opportunity for growth and move on to better, more interesting things:

“… *I look at that as being opportunity to move on to better things that are more interesting… So, it is not something that I consider to be a bad thing. I think there is something positive to come out of it*” (PC3).

Our findings agree with extant research; According to Langer (2014), stress is not a function of events but rather the view that each person takes of these events. An IT mindful individual, sensitive to different contexts and perspectives, does not rely on old and rigid categories but creates new ones depending on the context of the situation each time. As a result, IT mindfulness opens the views of individuals and disperses their stress, who do not perceive the possibility of losing their job to emerging technologies as something awful but rather as something inconvenient. By focusing on the advantages and opportunities that such a situation may bring, an IT mindful individual is able to accept it and be fine with it (Langer, 2014). As Langer (2014) highlights, there are no positive or negative outcomes but only different paths that we can choose, with each one of them including both challenges and opportunities.

Overall, our findings suggest that IT mindfulness may combat technostress experiences of individuals within workplace settings. By fostering sensitivity to different contexts, focus on the present moment, openness to novelty, new information and multiple perspectives, IT mindfulness can decrease the impact of technostress within the workplace. Moreover, our analysis revealed a “toolkit” of certain strategies that more IT mindful individuals deploy during technostress situations at work, thus yielding deeper insights into the relationship of IT mindfulness and technostress.

Regarding the practical implications of the current study, our findings offer a set of easy-to-follow and implement mindful strategies that corporate and HR managers can use and teach to staff in order to help employees cope more effectively with technostress conditions that arise daily within work settings. The revealed mindful strategies of the present study, such as prioritization of competing tasks and focus of attention on one task at a time, can be used as a set of techniques or else “toolkit” that employees can deploy daily at work as they are relevant to everyday work life of today’s organizational environments; situations where multiple incoming interruptions from several different ICT applications occur, information and email overload while an employee is working on a task or text and email communications happening after working hours. By adopting these strategies, knowledge workers can learn how to deal more effectively with distressing situations arising from technology usage, appropriate all the embedded benefits coming from using ICTs, such as increased productivity and performance, improve their work life while also contribute to the overall success of the organization. Further research is essential, in order to further evaluate the effectiveness of the proposed strategies in different types of organisations as well as different cultures.

## 6. Conclusion

Acknowledging the limited focus of previous technostress studies, this study contributes to the technostress literature and provides an enhanced understanding of this phenomenon by investigating ICT induced stress (technostress) from a mindfulness perspective. The current study adopted a mindfulness approach and examined it as technostress inhibitor that can alleviate the exposure of technostress within workplace settings. Overall, our findings suggest that mindfulness as well as IT mindfulness can protect individuals against the negative impact of stressful events that occur due to ICTs within the workplace. A more mindful individual is able to adapt and cope more effectively with technostress conditions that arise daily due to the extended use of organizational ICTs. As a result, a higher degree of mindfulness can alleviate the unsettling feelings of technostress. Either in the form of an intervention program embedded in the organizational settings or as a personal educational training, mindfulness can contribute to protecting as well as enhancing employees’ well-being while at the same time reduce workplace costs thus boosting the overall performance and success of the organization.

## 7. Limitations and further research

As with all empirical studies, the present study has certain limitations. At first, in this study the sample size was 10 participants as data saturation was reached at that point. Future studies should be employed to recruit a larger sample size and validate our results. Furthermore, in the current study participants show varying levels of mindfulness and IT mindfulness, averaging in both moderately. It would be interesting for future studies to recruit participants with very low/very high levels of mindfulness and compare our results. Also, the current study deployed interviews with knowledge workers to understand how mindfulness affects technostress experiences in the workplace, assessing their mindfulness levels using self-reported measures. It would be very interesting for future research to adopt a longitudinal approach in order to measure technostress before and after the implementation of mindfulness practices. As in the current study we have adopted the perspective of mindfulness as an inherent trait or quality, it would be very interesting to test the effects of mindfulness meditation on the phenomenon of technostress within the workplace. At last, in this study we have considered mindfulness and IT mindfulness as distinct concepts. Mindfulness is defined as a broad, more generic concept, explaining the behavior of an individual in various contexts of everyday life. On the other hand, IT mindfulness is defined as an IT specific trait, evident only when working with technology and oriented in the IT context. Thus, one person can be mindful but not necessarily highly IT mindful. There is very limited previous research suggesting that the two concepts constitute distinct entities, thus future research is essential in examining in more depth the relationship between the two concepts, establishing their similarities and differences.

## Data availability statement

The raw data supporting the conclusions of this article will be made available by the authors, without undue reservation.

## Ethics statement

The studies involving humans were approved by Brunel University London, United Kingdom. The studies were conducted in accordance with the local legislation and institutional requirements. The participants provided their written informed consent to participate in this study.

## Author contributions

AI contributed to the conception and design of the study, performed the statistical analysis, and writing the draft of the manuscript.
