# Supplementary material for: Mindfulness and technostress in the workplace: a qualitative approach
Source: Front Psychol. 2023 Nov 13;14:1252187. doi: 10.3389/fpsyg.2023.1252187 (PMC10679397; doi:10.3389/fpsyg.2023.1252187)
Supplement: Supplementary file 1 [file Presentation_1.pdf]

frontiers

Appendix

Scenarios used in semi-structured interviews

1. ***<u>Scenario 1: Techno Overload</u>***
- How often do you read/check your email at work?
- When you get an email (at work), how soon do you feel you need to respond?
- Can you describe your multitasking during a regular working day?
- In your opinion, is it easier or harder to focus on the task at hand when you are engaged in other tasks at the same time?
- Sometimes, when you are at work, your phone rings, one/several email comes in and a colleague is asking for your help while you are working on a task/project. Can you please describe in detail a similar scenario in your situation?
- How do you usually respond in such a case?
- Is this scenario something that happens often?
- How did you feel in this situation?

2. ***<u>Scenario 2: Techno invasion</u>***
- Are you expected to be contactable (approachable) outside working hours?
- How do you feel about this?
- Do you have time to unplug from your job completely?
- Sometimes you receive emails about work related issues, outside regular working hours, at night, during a weekend or your annual leave. Can you please describe in detail a similar scenario in your situation and how you usually respond in such a case?
- How did you feel in this situation

3. ***<u>Scenario 3: Techno Complexity</u>***
- Are you expected to stay current with technological advances in your domain?
- Do you have any examples to share?
- Sometimes companies decide to update technologies (operating systems, information systems, email clients) and as a result you need time to 'learn' how to use the new, more advanced, technology that you will be using for your daily work tasks. During this period, you might encounter errors & problems with the new applications, they might be running slow and/or crash while you are working on a task/project. Can you please describe in detail a similar scenario in your situation?
- How do you usually respond in such a case?
- How did you feel in this situation?

4. ***<u>Scenario 4: Techno insecurity</u>***
- Sometimes employees in certain positions in a company, such as Social Media Managers, get replaced by newer, younger, more technological skilled people who have a higher level of

competence with technology and are more enthusiastic to use new technologies. Is that a common encounter in your company?

- Do you have any examples to share?
- Some people believe that in the near future employees will be replaced by emerging technologies. What is your opinion? How do you feel about this?
